# Triglyceride–Glucose Index and New-Onset Type 2 Diabetes Mellitus in Middle-Aged Men

**DOI:** 10.3390/metabo15080537

**Published:** 2025-08-08

**Authors:** Lanfranco D’Elia, Domenico Rendina, Roberto Iacone, Pasquale Strazzullo, Ferruccio Galletti

**Affiliations:** Department of Clinical Medicine and Surgery, “Federico II” University of Naples Medical School, Via S. Pansini 5, 80131 Naples, Italy; domenico.rendina@unina.it (D.R.); iacone@unina.it (R.I.); strazzul@unina.it (P.S.)

**Keywords:** triglyceride–glucose index, insulin resistance, diabetes, cardiovascular risk

## Abstract

**Background:** Type 2 diabetes mellitus (T2DM) is a major contributor to cardiovascular disease worldwide, with insulin resistance (IR) being a central pathophysiological mechanism. The triglyceride–glucose (TyG) index, derived from routine fasting measures, has emerged as a simple yet reliable proxy for IR and is increasingly recognised for its prognostic value in metabolic disorders. Despite growing interest, longitudinal evidence on TyG and incident T2DM, especially within European cohorts, remains limited and inconsistent. Therefore, we aimed to estimate the predictive role of TyG on the development of T2DM in an 8-year follow-up observation of a sample of adult men. **Methods:** We analysed data from 789 adult men without baseline T2DM, enrolled in the Olivetti Heart Study. Receiver operating characteristic curve analysis was used to determine the optimal TyG threshold for predicting new-onset T2DM. **Results**: Baseline TyG exhibited a strong, linear association with the subsequent development of T2DM. A TyG value above 4.88 was associated with an approximately twofold increase in risk, both before and after adjustment for confounding factors. **Conclusions**: The principal findings of this study indicate a significant predictive value of the TyG index in the development of new-onset T2DM. These observations suggest that the TyG index may serve as a low-cost, simple, and non-invasive tool for early cardio-metabolic risk assessment.

## 1. Introduction

Type 2 diabetes mellitus (T2DM) is a global epidemic and a leading modifiable risk factor for cardiovascular morbidity and mortality [1]. Individuals with T2DM exhibit markedly elevated rates of cardiovascular disease [2], highlighting the critical need to identify high-risk individuals prior to the onset of overt hyperglycaemia. Insulin resistance (IR), the pivotal metabolic abnormality that precedes and propels T2DM, is the mechanistic link between altered glucose levels and cardiovascular injury [3]. Accordingly, early detection of IR is crucial for stratifying individuals who are at high cardiovascular risk and for guiding timely preventive interventions. Various markers have been evaluated to assess IR [4]. The hyperinsulinemic–euglycemic clamp is regarded as the gold-standard technique [4]; however, its technical complexity, duration, and cost render it impractical for routine clinical or large-scale epidemiological use. Several surrogate indices are based on the measurement of fasting insulin, which limits their feasibility for large observational studies and routine healthcare settings. To overcome these constraints, the triglyceride–glucose (TyG) index has been proposed as an alternative marker [5,6]. Derived from routine fasting triglyceride and plasma glucose measurements, the TyG index is simple, inexpensive, and readily applicable in both clinical practice and population-based research. TyG correlates strongly with IR as quantified by the clamp technique and with the Homeostatic Model Assessment of IR (HOMA-IR) index, the principal methodological instrument currently adopted in epidemiological studies and clinical practice [7]. Moreover, where the HOMA-IR index primarily reflects hepatic IR, the TyG index captures both hepatic and peripheral IR [8]. Its excellent reproducibility, lack of reliance on insulin assays, and compatibility with automated laboratory panels make the TyG index an attractive candidate for widespread risk assessment. Epidemiological studies suggest a substantial direct association between TyG and cardiovascular risk in different settings [8,9,10,11,12,13,14]. In this context, heterogeneous data indicate the TyG index as a predictor of incident T2DM [15,16,17,18,19,20]. Meta-analyses of predominantly East Asian cohorts indicate that individuals in the highest TyG strata have more than double the risk of developing T2DM compared to those in the lowest strata [21,22]. However, only two European cohorts [23,24] have examined the TyG–T2DM association, and those studies did not assess the shape of the association (i.e., linear or non-linear) and define optimal cut-offs.

Therefore, in consideration that one of the global targets for non-communicable diseases is to reduce the incidence of T2DM [1], given the crucial role of early markers of risk of T2DM to achieve this target, and the limited epidemiological data delineating the association between TyG and the development of T2DM in European populations [23,24], we explored the prognostic value of baseline TyG levels for predicting the development of T2DM over an 8-year follow-up in a population-based cohort of men participating in the Olivetti Heart Study.

## 2. Materials and Methods

### 2.1. Study Population

The Olivetti Heart Study was a prospective occupational cohort study conducted among male employees in Southern Italy [25,26]. The design, implementation, and reporting of the study adhered to the STrengthening the Reporting of OBservational studies in Epidemiology (STROBE) guidelines [27] (Supplemental Table S1). A total of 1085 adult participants were initially assessed between 1994 and 1995, and 84% underwent re-evaluation during the follow-up phase conducted between 2002 and 2004.

For the present analysis, individuals were excluded sequentially for the following reasons: incomplete data from either examination (*n* = 50), and the presence of T2DM at baseline (defined as fasting blood glucose ≥ 126 mg/dL or current use of glucose-lowering therapy) (*n* = 72). The final analytical cohort comprised 789 participants.

### 2.2. Examination Procedures

A thorough description of the methodological framework employed in the Olivetti Heart Study has been extensively reported in the previously published peer-reviewed literature [25,26]. At both baseline and follow-up visits, physical examinations were conducted between 08:00 and 11:00 in a quiet, temperature-controlled setting following a minimum 13-hour fast. Participants were permitted to continue their normal daily activities but were explicitly advised to avoid vigorous exercise. They were also instructed to abstain from smoking and from consuming alcohol, coffee, tea, or other caffeinated beverages from the evening prior to the visit. The baseline visit comprised a clinical assessment, anthropometric measurements, and a fasting venous blood sample, followed by a timed urine collection. Participants also completed a comprehensive questionnaire covering medical history, occupational and leisure-time physical activity, smoking status, and alcohol consumption.

A fasting venous blood sample was collected from each participant. Blood specimens were immediately centrifuged and stored at −70 °C until biochemical analyses were performed. Serum glucose and triglyceride concentrations were determined using automated enzymatic methods (Cobas-Mira, Roche Diagnostics, Italy). T2DM was defined according to standardised criteria (fasting blood glucose level ≥ 126 mg/dL or current anti-T2DM therapy) [28], both at baseline and follow-up examinations. The TyG index was calculated using the following formula: Ln [triglycerides (mg/dL) × fasting glucose (mg/dL)]/2 [5]. Serum creatinine levels were measured by the picric acid colorimetric method. The estimated glomerular filtration rate (eGFR) was computed using the Chronic Kidney Disease Epidemiology Collaboration (CKD-EPI) 2009 equation [29].

Body weight and height were assessed using a calibrated beam balance scale with a vertical stadiometer. Body mass index (BMI) was calculated as weight in kilograms divided by height in metres squared (kg/m^2^); excess body weight was defined as a BMI ≥ 25 kg/m^2^ [30]. Waist circumference (WC) was measured at the level of the umbilicus with the participant standing upright, using a non-extensible plastic measuring tape. Abdominal obesity was defined as a WC > 102 cm [31].

Participants were stratified into two groups based on alcohol consumption: those consuming at least one glass of wine (or an equivalent amount of other alcoholic beverages) per day, and those reporting no alcohol intake. Physical activity was categorised according to whether participants engaged in habitual aerobic exercise for at least 30 min per day.

### 2.3. Statistical Analysis

All statistical analyses were performed using SPSS software (version 29, SPSS Inc., Chicago, IL, USA) and the statistical package R (version 4.3.1). As baseline values of eGFR displayed skewed distributions, logarithmic transformation was applied before statistical analysis. Bivariate associations between TyG and the variables under investigation were assessed using Pearson’s correlation coefficients. Differences in group means were evaluated using analysis of variance, while associations between categorical variables were examined by the Chi-squared test.

To explore the functional form of the association between TyG (modelled as a continuous variable) and the risk of T2DM, restricted cubic spline (RCS) regression models with four knots located at the 5th (reference), 35th, 65th, and 95th percentiles were employed [32]. Given the linear relationship between TyG and new-onset T2DM, binary logistic regression analysis was used to estimate the predictive role of baseline TyG (as a continuous variable) on the development of T2DM. The impact of traditional risk factors and that of potential confounding factors of the sample (*p*-value < 0.2, relating to the comparison between those who developed T2DM and not) was explored by multivariate models adjusted for baseline age, BMI or WC, renal function, physical activity, alcohol consumption, and lipid-lowering therapy.

Receiver operating characteristic (ROC) curve analysis was performed to assess the discriminatory power of the TyG index in identifying individuals who developed T2DM during follow-up. The area under the curve (AUC), together with its 95% confidence interval (CI), was calculated. The optimal cut-off value for TyG (4.88) was determined using Youden’s index. Participants were subsequently stratified into two groups based on this threshold: High-TyG (TyG > 4.88) and Low-TyG (TyG ≤ 4.88). Binary logistic regression analysis was employed to evaluate the association between baseline TyG status (dichotomised) and the risk of incident T2DM, adjusting for main potential confounders as specified above.

The results are reported as percentages, as mean with standard deviation (SD), or as an odds ratio (OR) and a 95% CI (Bootstrap CI, 1000 iterations). A two-tailed *p*-value of less than 0.05 was considered indicative of statistical significance.

## 3. Results

The baseline characteristics of the total sample (*n* = 789) are reported in Table 1.

The mean age at baseline was 51.3 years; 58.8% of the participants were overweight, 14.9% were obese, 16.9% had abdominal obesity, 79.7% consumed alcohol, 35% engaged regularly in physical activity, and 12% used lipid-lowering therapy.

At baseline, TyG was significantly and directly correlated with BMI (r = 0.19) and WC (r = 0.19), and inversely correlated with renal function (r = −0.11).

At the eight-year follow-up, the overall new-onset T2DM rate was 6.8%. Baseline TyG was significantly greater in those who developed T2DM at follow-up than in those who did not (TyG, 4.82 + 0.29 vs. 4.71 + 0.25, *p* = 0.002). In addition, those who developed T2DM had a significantly higher baseline body weight, WC, and percentage of ongoing lipid-lowering therapy (Table 2).

**Figure 1:**
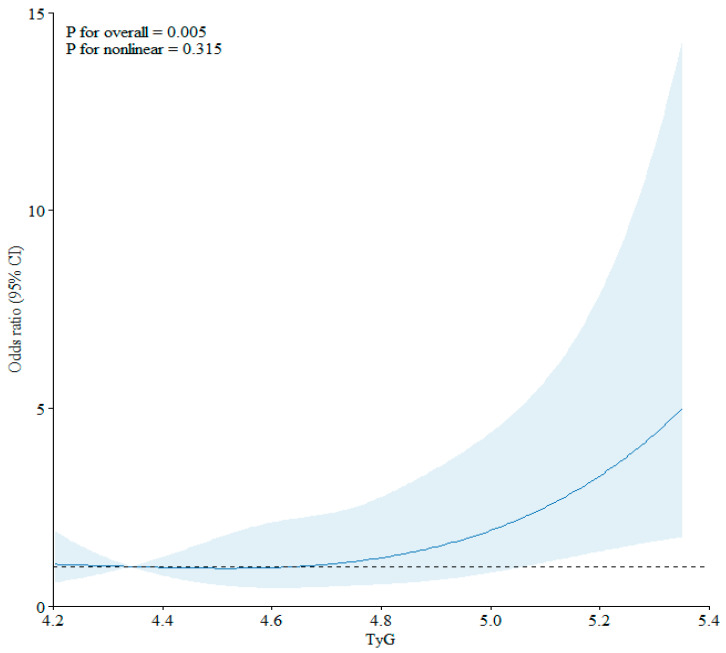
Association between the triglyceride–glucose (TyG) index and risk of new-onset type 2 diabetes mellitus using a restricted cubic spline regression model. Solid lines indicate odds ratios, and shadow shapes indicate 95% confidence intervals (CIs).

The RCS regression model detected a linear relationship between TyG and new-onset T2DM (test for non-linearity: *p* = 0.315) (Figure 1).

Logistic regression analysis revealed a statistically significant association between baseline TyG levels and the risk of T2DM at follow-up (for a 1-Unit increase in baseline TyG, OR: 5.19, 95% CI: 1.78–15.07, *p* = 0.002). The independent predictive value of the TyG index remained statistically significant after adjustment for the principal confounding variables (for a 1-Unit increase in TyG: model including BMI, OR: 3.51, 95% CI: 1.12–11.01; model including WC, OR: 3.41, 95% CI: 1.07–10.81).

**Figure 2:**
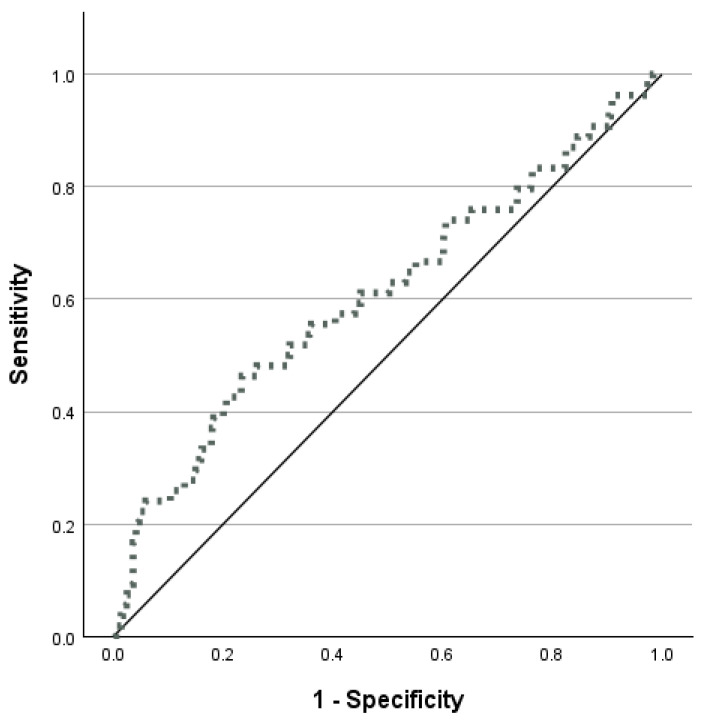
Receiver-operating characteristic (ROC) curve analysis for the triglyceride–glucose (TyG) index.

The AUC for the relationship between TyG and new-onset T2DM showed a significant ability to detect T2DM (AUC and 95% CI: 0.61, 0.57–0.64, *p* = 0.004; cut-off point: 4.88; sensitivity: 46%, specificity: 76%; positive predictive value: 12%; negative predictive value: 95%) (Figure 2).

Based on the stratification using the optimal cut-off point identified by ROC curve analysis, we assessed the predictive value of the threshold. As expected, the proportion of participants who developed T2DM was significantly higher in the High-TyG group compared to the Low-TyG group (12.4% vs. 4.9%, *p* < 0.001).

The High-TyG group had a significantly higher baseline BMI, WC, and percentage of ongoing lipid-lowering therapy, and a lower eGFR than the Low-TyG group, while no significant difference was detected for age, physical activity, and alcohol consumption (Table 3).

Logistic regression analysis demonstrated a statistically significant positive association between elevated TyG levels and the risk of the incident of T2DM, both in unadjusted models (High-TyG vs. Low-TyG, OR: 1.88, 95% CI: 1.22–2.89) and following adjustment for principal confounding variables (model including BMI: OR: 2.33, 95% CI: 1.29–4.23, Wald test: TyG = 8.4 vs. BMI = 6.1; model including WC: OR: 2.32, 95% CI: 1.27–4.24, Wald test: TyG = 9.6 vs. WC = 2.3).

## 4. Discussion

To the best of our knowledge, this is the first study to delineate the form of the association between the TyG index and the incidence of T2DM in a European population. The key findings reveal a positive and linear relationship between TyG and the risk of new-onset T2DM in a cohort of middle-aged men drawn from an unselected working population. Importantly, TyG values exceeding 4.88 were associated with an approximate twofold increase in T2DM risk, independent of major confounding factors, including age and anthropometric parameters.

Our results reinforce the robustness of the association between TyG and new-onset T2DM, regardless of whether the index was analysed as a categorical or continuous variable, and in fully adjusted models. Moreover, although the discriminatory capacity of TyG in this cohort was limited by relatively low performance metrics, the stratification according to the threshold identified a subgroup (i.e., High-TyG) that was characterised by markedly adverse cardio-metabolic profiles, in line with previous evidence [33,34]. The observed linear relationship between TyG and the risk of T2DM further supports the notion of a potentially more consistent and reliable predictive capacity. Hence, the TyG index may serve as a feasible and cost-effective marker of early cardio-metabolic risk, particularly within the context of large-scale epidemiological investigations or routine clinical practice, where access to more complex or resource-intensive assays may be limited.

The TyG index also showed a predictive value that was independent of conventional anthropometric markers. At baseline, it exhibited modest yet statistically significant positive correlations with both BMI and WC (r = 0.19 for both). Notably, the association between the TyG index and incident T2DM remained robust following adjustment for either parameter. Moreover, the TyG index showed a stronger association with incident T2DM than either BMI or WC alone, as evidenced by comparative statistical analyses. These findings suggest that the TyG index reflects additional features of metabolic dysregulation not captured by general or central measures of adiposity. Therefore, the TyG index may represent a valuable adjunctive biomarker for the early identification and risk stratification of T2DM in both clinical and epidemiological contexts.

Our findings are consistent with, and extend, previous research evaluating TyG as a predictor of T2DM [21,22,23,24]. Two meta-analyses reported a significant positive association between higher TyG values and increased risk of incident T2DM [21,22]. Notably, only one of these studies assessed the dose–response relationship and identified a non-linear association [22], while the other did not examine the shape of the relationship [21]. Both meta-analyses reported considerable heterogeneity and evidence of potential publication bias, which may have attenuated the robustness of the pooled estimates. Variability in sample characteristics, including ethnicity, sex distribution, genetic and cardio-metabolic profiles, and comorbidities, may also account for some inconsistencies with our findings.

To date, only two studies have investigated this relationship in European cohorts. The Spanish CUN-vascular cohort (*n* = 4820) confirmed the predictive value of TyG over 8 years of follow-up, but included only normoglycemic individuals, employed quartile-based stratification, did not assess the shape of the association, did not identify an optimal TyG threshold, and did not adjust for lipid-lowering therapy [24]. Similarly, data from the Rotterdam Study (*n* = 9564) demonstrated a significant association, particularly among women, when stratified by quartiles. However, the study did not characterise the functional nature of the association nor determine evidence-based threshold values to support clinical interpretation [23].

The reliability of the present findings is supported by the following key methodological and analytical strengths: (1) the soundness of the results; (2) the prospective design with the relatively long length of follow-up; (3) the careful standardisation of data collection at both examinations; (4) the assessment of the shape of the association between the baseline TyG and the development of T2DM; (5) the statistical determination of an optimal discriminatory threshold using ROC curve analysis; (6) the independent role of TyG from the effect of therapy, because participants with anti-T2DM therapy at baseline were excluded from the analysis, and the effect of lipid-lowering therapy at baseline was considered in the multivariate models.

Nonetheless, the study has some limitations: (1) The observational design of the study does not allow us to establish a causal relationship, but the longitudinal design permits the temporal sequence of events to be established. (2) The participation of only adult white male individuals limits the generalisability of our findings. Sex-specific hormonal, behavioural, and body composition differences may influence the TyG–T2DM association. For instance, women tend to accumulate more subcutaneous fat and exhibit different patterns of insulin sensitivity compared to men [35,36,37]; thus, further studies in women and more ethnically diverse populations are warranted to assess whether similar associations and cut-offs apply. (3) The potential influence of unmeasured variables cannot be entirely ruled out, as, for instance, dietary factors such as potassium intake, strongly associated with IR and the risk of T2DM [38,39,40,41], were not systematically assessed. (4) Another limitation of the present study is the lack of a direct comparison between the TyG index and the hyperinsulinemic–euglycemic clamp, which is considered the reference standard for assessing IR. Nonetheless, the TyG index has been previously validated against this gold-standard method [6]. Furthermore, in contrast to more complex, resource-intensive, and costly techniques requiring insulin assays, the TyG index offers a pragmatic, reproducible, and cost-effective alternative, relying solely on routinely available biochemical parameters. These characteristics enhance its applicability in large-scale epidemiological studies, particularly in clinical settings where resources are limited.

## 5. Conclusions

The main findings of this study indicate that TyG is linearly and positively associated with new-onset T2DM in middle-aged men. In particular, TyG values higher than 4.88 were associated with an approximately doubled risk of new-onset T2DM, independent of the main potential confounders. The findings show the robustness of the association between TyG and incident T2DM when analysed as both the categorical and continuous variable, independently of main confounders. In light of these findings, and considering that T2DM is a major risk factor for cardiovascular disease, we conclude that the TyG index may serve as an unexpansive, simple, and non-invasive adjunctive tool for early cardio-metabolic risk stratification, particularly in large-scale population-based or epidemiological contexts.

Future investigations should focus on validating these results across diverse populations and age groups, refining the optimal cut-off values for risk discrimination, and evaluating the integration of TyG into comprehensive, multifactorial risk prediction models. Additionally, longitudinal studies are warranted to determine whether TyG-guided interventions can effectively reduce the incidence of T2DM and related complications. Finally, implementation research should assess TyG screening in real-world settings, especially in resource-constrained and low- to middle-income regions, where simplicity and cost-effectiveness are critical. Such studies would clarify the feasibility of using the TyG index as a practical tool in public health programmes, bridging the gap between observational insight and actionable prevention. Ultimately, these lines of inquiry may help to position the TyG index not only as a robust predictor, but also as an intervention-responsive biomarker within personalised and population-level strategies for metabolic disease prevention.

## Figures and Tables

**Table 1:** Baseline characteristics of the study participants.

N. of Participants	789
Age (yrs)	51.3 ± 7.2
BMI (kg/m^2^)	26.9 ± 3.0
Excess body weight (%)	73.7
Waist Circumference (cm)	94.4 ± 8.4
Abdominal obesity (%)	16.9
eGFR (mL/min/1.73 m^2^) ^1^	97.7 ± 1.2
Renal damage (eGFR < 60 mL/min/1.73 m^2^) (%)	0.5
Lipid-lowering therapy—yes (%)	12.0
Physical activity—yes (%)	35.0
Alcohol consumption—yes (%)	79.7
TyG (Units)	4.72 ± 0.25

Data are expressed as means ± SD or as percentages; BMI: body mass index; eGFR: estimated glomerular filtration rate; TyG: triglyceride–glucose index. ^1^ Data expressed as geometric mean.

**Table 2:** Baseline features of the study participants stratified by incident of type 2 diabetes mellitus (T2DM).

Incident T2DM	YES	NO	*p*-Value
N. of participants	54	735	
Age (yrs)	51.9 ± 5.6	51.2 ± 7.3	0.5
BMI (kg/m^2^)	28.4 ± 2.9	26.8 ± 3.0	<0.001
Excess body weight (%)	90.7	9.3	<0.001
Waist Circumference (cm)	98.0 ± 8.1	94.2 ± 8.3	0.001
Abdominal obesity (%)	28.3	16.1	0.02
eGFR (mL/min/1.73 m^2^) ^1^	95.5 ± 1.1	97.7 ± 1.2	0.7
Renal damage (eGFR < 60 mL/min/1.73 m^2^) (%)	0	0.5	0.6
Lipid-lowering therapy—yes (%)	25.9	11.0	0.001
Physical activity—yes (%)	43.4	34.4	0.2
Alcohol consumption—yes (%)	73.6	80.1	0.2
TyG (Unit)	4.82 ± 0.29	4.71 ± 0.25	0.002

Data are expressed as means ± SD or as percentages; BMI: body mass index; eGFR: estimated glomerular filtration rate; TyG: triglyceride–glucose index. ^1^ Data expressed as geometric mean.

**Table 3:** Baseline features of the study participants stratified by the triglyceride–glucose (TyG) index threshold (*n* = 789).

	High-TyG (>4.88)	Low-TyG (≤4.88)
N. of participants	202	587
Age (yrs)	51.6 ± 6.8	51.2 ± 7.3
BMI (kg/m^2^)	27.7 ± 2.8 *	26.6 ± 3.0
Excess body weight (%)	83.2 *	70.5
Waist circumference (cm)	97.1 ± 7.7 *	93.5 ± 8.4
Abdominal obesity (%)	24.0 *	14.5
eGFR (mL/min/1.73 m^2^) ^1^	94.8 ± 1.1 *	97.7 ± 1.2
Renal damage (eGFR < 60 mL/min/1.73 m^2^) (%)	0	0.5
Lipid-lowering therapy—yes (%)	19.8 *	9.4
Physical activity—yes (%)	36.7	34.4
Alcohol consumption—yes (%)	80.6	79.4
TyG (Unit)	5.0 ± 0.1 *	4.6 ± 0.2

Data are expressed as means ±SD or percentages; BMI: body mass index; eGFR: estimated glomerular filtration rate; TyG: triglyceride–glucose index. * High-TyG vs. Low-TyG: *p* < 0.05. ^1^ Data expressed as geometric mean.

## Data Availability

Some or all datasets generated and/or analysed during the current study are not publicly available but are available from the corresponding author on reasonable request.

## Supplementary Materials

The following supporting information can be downloaded at https://www.mdpi.com/article/10.3390/metabo15080537/s1: Table S1: STROBE Statement—Checklist of items that should be included in reports of cohort studies.

## Abbreviations

AUC	Area under the curve
BMI	Body mass index
eGFR	estimated glomerular filtration rate
HOMA	Homeostatic Model Assessment
IR	Insulin resistance
OHS	Olivetti Heart Study
RCS	Restricted cubic splines
ROC	Receiver-operating characteristic
STROBE	STrengthening the Reporting of OBservational studies in Epidemiology
T2DM	Type 2 Diabetes Mellitus
TyG	Triglyceride–glucose index
WC	Waist circumference
